# Investigating the Mechanical Properties of Annealed 3D-Printed PLA–Date Pits Composite

**DOI:** 10.3390/polym15163395

**Published:** 2023-08-13

**Authors:** Ahmed Fouly, Thamer Albahkali, Hany S. Abdo, Omar Salah

**Affiliations:** 1 Mechanical Engineering Department, College of Engineering, King Saud University, P.O. Box 800, Riyadh 11421, Saudi Arabia; talbahkali@ksu.edu.sa; 2 The King Salman Center for Disability Research, Riyadh 11421, Saudi Arabia; 3 Department of Production Engineering and Mechanical Design, Faculty of Engineering, Minia University, Minia 61519, Egypt; 4 Center of Excellence for Research in Engineering Materials (CEREM), King Saud University, P.O. Box 800, Riyadh 11421, Saudi Arabia; habdo@ksu.edu.sa; 5 Mechanical Design and Materials Department, Faculty of Energy Engineering, Aswan University, Aswan 81521, Egypt; 6 Mechatronics Engineering Department, Faculty of Engineering, Assiut University, Assiut 71515, Egypt; omar.salah@aun.edu.eg

**Keywords:** PLA green composite, 3D printing, ANFIS, artificial intelligent, rehabilitation medicine

## Abstract

Biomedical applications are crucial in rehabilitation medicine, assisting individuals with disabilities. Nevertheless, materials failure can sometimes result in inconvenience for users. Polylactic Acid (PLA) is a popular 3D-printed material that offers design flexibility. However, it is limited in use because its mechanical properties are inadequate. Thus, this study introduces an artificial intelligence model that utilizes ANFIS to estimate the mechanical properties of PLA composites. The model was developed based on an actual data set collected from experiments. The experimental results were obtained by preparing samples of PLA green composites with different weight fractions of date pits, which were then annealed for varying durations to remove residual stresses resulting from 3D printing. The mechanical characteristics of the produced PLA composite specimens were measured experimentally, while the ANSYS model was established to identify the composites’ load-carrying capacity. The results showed that ANFIS models are exceptionally robust and compatible and possess good predictive capabilities for estimating the hardness, strength, and Young’s modulus of the 3D-printed PLA composites. The model results and experimental outcomes were nearly identical.

## 1. Introduction

Over time, the field of orthotics has advanced to allow additional support for individuals with disabilities in the performance of daily activities. For example, in sports, orthotics have progressed to meet the demands of people for their post-injury recovery [1]. Knee joint injuries are usually treated using knee orthotics. Structural knee orthotics are considered the most effective treatment for such injuries, but they come with a high cost ranging from $100 to $900 per brace, which makes the recovery process very expensive [2]. The main reason for such a problem is the high cost of materials used in structural orthotics, coupled with production process costs. To solve such a problem, biomedical scientists try to develop new materials with acceptable properties to be used in orthotics. Such materials should possess sufficient mechanical properties to withstand different loading conditions. This can be achieved by utilizing advanced simulation and characterization tools to optimize the performance of new materials and manufacturing techniques.

Recently, polymers have attracted the attention of many scientists because they have many unique advantages [3]. Researchers have used polymers in many applications and fields, for example, electronics, biophysics, prosthetics, vehicles, medicine, and sensors [4]. Polymers should possess biostability and biodegradability properties in biomedical applications, making them biomedical polymers or biopolymers [5]. In biomedical applications, polymers were first utilized by a British ophthalmologist who fabricated intraocular lenses based on a famous biomedical polymer, which today is called PMMA [6]. As research on polymers in biomedical applications has progressed, a library of polymeric materials was established in which different polymers were categorized based on their specific biomedical applications and functions. This library allows researchers to easily access information on the properties and performance of various polymers and to select the most suitable polymer for a given application. The library also helps to promote the development of new polymeric materials with improved properties and performance for biomedical applications.

The additive manufacturing technique that uses 3D printers is an attractive way to create complex 3D structures and has been used by researchers to develop various biomedical parts. Examples include parts for oral and maxillofacial surgery [7], human organs [8], electronic ears [9], and femoral implant rods [10]. Three-dimensional printing technology can work with many types of polymers, but Polylactic Acid (PLA) is considered the prominent material used with this technique due to its low warping issues, environmental friendliness, biocompatibility [11], and ease of production and use [12]. However, its low stiffness and strength are a barrier to its use in commercial applications [13]. Researchers have attempted to overcome these limitations by blending PLA with other polymers, incorporating fillers, and using advanced processing techniques to improve its different properties [14]. Researchers in the field of biomedical polymers have been exploring the utilization of natural fillers instead of synthesized ones to adapt the properties of the materials while maintaining their biocompatibility and environmental friendliness.

Natural fillers can be sourced from materials found in nature without any cost. Researchers have investigated using materials such as Paulownia wood and Osage orange wood in combination with PLA and found that it increases elongation and stiffness but causes a decrease in tensile strength [15]. Another study looked at the impact of adding varying amounts of beechwood, up to 50%, to 3D-printed PLA and found that increasing the wood content led to an increase in the surface roughness as well as the existence of many voids and wood debris; it also caused a deterioration in the storage modulus while the glass transition temperature underwent no change [16]. Liu et al. [17] evaluated the influence of incorporating several natural materials, newspaper, eucalyptus, lignin, pine, and pulp into PLA at different concentrations. They evaluated the resulting composites’ print quality, melt flow index, and mechanical properties. They recorded a 74% enhancement in the strength associated with the addition of 15% lignin. Ahmed et al. [18] examined the effect of utilizing date pits particles as a filler inside the PLA with a variation in the date pits concentrations of 0–10 wt.%. They found a slight enhancement in the hardness and strength of the PLA-produced composites, and they refer to not using any modification or additional processes for the composite after printing.

According to previous research, 3D-printed products often have poor mechanical attributes, regardless of the PLA or other materials that may be utilized in the 3D printing technology [19]. To understand these weaknesses, it is essential to consider the material’s performance during and after the printing process. The interaction between two printed filaments on the bed of the printer goes through different stages: the surfaces of the filaments come into contact; then, a neck forms; next, there is diffusion at the interface; and finally, the structure becomes randomized. This process results in many voids in the bond between the filaments, which can weaken the strength of the 3D-printed part [20]. Additionally, the temperature between the filament already laid on the bed and the new one coming from the nozzle is different. This difference generates internal stresses that can cause different characteristics in the printed product.

The utilization of heat treatment is a common practice in the metal industry to control and manipulate the properties of metallic materials. The reduction in residual stress generated throughout the samples’ fabrication is considered the main goal of the heat treatment process. Similarly, as previously discussed, during the 3D printing process, internal stresses are generated within the product, negatively impacting its properties. As a result, researchers have suggested that heat treatment could also be applied to polymers, and not just be limited to metals [21]. Bhandari et al. [22] attempted to use heat treatment on a PLA–carbon composite. The results showed a noticeable improvement in the composite’s mechanical characteristics compared with pure PLA. Jayanth et al. [23] investigated the mechanical characteristics of heat-treated PLA parts, applying 0, 90, 100, and 120 °C to the samples. They found an 80% increase in tensile properties by annealing at 100 °C and a 73% improvement in heat resistance. Though these results demonstrate the effectiveness of heat treatment on polymers, the current knowledge of its impact on 3D-printed products is limited. Therefore, more research is needed to provide reliable and predictive information on the behavior of PLA and its composites.

As previously mentioned, the 3D-printed sample characteristics are influenced by various parameters that can be determined before and after printing. Researchers have attempted to develop models that can link the changes in these parameters (inputs) to the performance of the 3D-printed products (outputs) [24]. An adaptive neuro-fuzzy inference system (ANFIS) is an artificial intelligence technique that can be combined with neural networks to create input–output relationships [25,26]. ANFIS is considered a superior option for estimating input–output relationships in nonlinear situations [27], which is particularly relevant regarding parameters that affect 3D-printing technology. Ibrahim et al. [28] aimed to predict the rate at which polytetrafluoroethylene composites wear due to factors such as the reinforcement weight fraction, the density of the composite, and the sliding distance. They compared the predictions made by three models: one based on multilinear regression, another on a feed-forward neural network, and a third on ANFIS. They found that the ANFIS model was 45.8% more accurate than the model using multilinear regression.

The current study aims to develop an artificial intelligence model using ANFIS to estimate the mechanical properties of PLA green composites used in biomedical applications. To achieve that, PLA is mixed with date pit particles (0%, 2%, 4%, 6%, 8%, and 10% by weight) to create the green composite. The samples are then annealed at 100 °C for various duration times (0, 2.5, 5, 10, and 20 h) to prevent the formation of voids and reduce residual stress throughout the 3D-printing procedures. The mechanical performance of unannealed and annealed PLA-DP composites were evaluated experimentally. ANSYS was used to create a model knee orthotics to estimate the stress generated due to the human load. Finally, ANFIS models were developed using MATLAB that could predict the mechanical performance of the PLA composites with a change in different production parameters such as weight fraction of DP or annealing time.

## 2. Experimental Work Description

### 2.1. Composite Specimens Preparation

Two materials were primarily used in the current study, PLA and date pits. The PLA was procured from China (company name: Shanghai Nuolei CNC Router Equipment). The material data sheet indicates that the PLA is made from corn starch, resulting in minimal shrinkage and warping. Furthermore, it is made of 100% new raw material and is considered an eco-friendly material. The PLA was provided as filaments with a diameter of 1.75 mm, suitable for printing in the range of 190 to 220 °C. The date pits came in their raw nature from Al Khmash Company, Kingdom of Saudi Arabia. Date fruits come in various types, and their characteristics may vary. The date pits used in this study were extracted from Kholas dates. Previous research has shown that the seeds of Kholas dates consist of 4.8–6.9% protein, 2.4–4.7% carbohydrates, 8.6–12.5% moisture, 67.6–74.2% dietary fiber, 5.7–8.8% lipids, and 0.8–1.1% ash [29].

The date pits were first rinsed inside a water bath to prevent any remaining contaminants, and remaining date molasses were used to prepare the date pit powder. The seeds were then left to dry for one month in the sun to eliminate any moisture. Thereafter, they were dried again in an oven (JSV0-60T) for one week at 60 °C. The date pits were then reduced to powder size in three stages: crushing with a mortar and pestle, pulverization in a grain miller, and finally, milled in a ball milling machine for 8 h. Once the grinding process was completed, the obtained powder was dried for 24 h in an oven to remove any remaining moisture.

**Figure 1:**
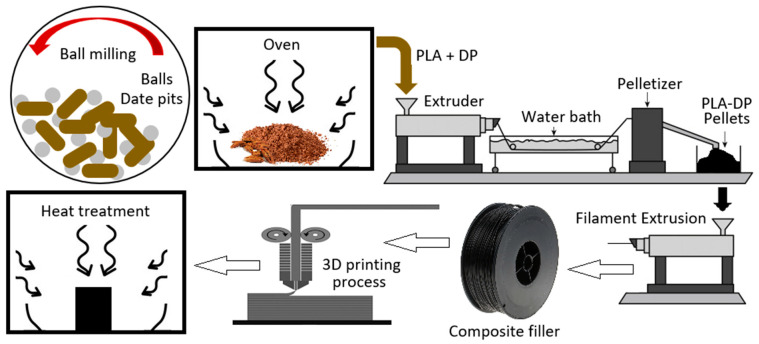
PLA–DP composites’ preparation.

Creating the PLA composites began by taking PLA filament and running it through a pelletizer to create small PLA pellets. These pellets were then weighed and combined with date pit powder in varying amounts, with the weight fraction of date pit particles increasing in 2% increments from 0 to 10%. The PLA pellets and the date pit particles were mixed using a stirrer for 20 min at room temperature at a speed of 300 rpm. This ensured that the date pit powder was homogeneously distributed throughout the PLA matrix. The mixture was then processed through a twin extruder that had many temperature regions starting from 160 °C up to 210 °C and the extruder speed was set to 50 rpm. The screw of the extruder dimensions were 16 mm diameter and had a length-to-diameter ratio 40 L/D. The blend after extrusion from the capillary dye was cooled in a water sink and then pelletized one more time. To ensure date pit distribution inside the PLA, the mixed pellets were then run multiple times through the twin extruder. The mixing process was repeated three times, for each composition, without the addition of any adhesion materials. To produce filaments that are suitable to be used in 3D printers, a Filabot EX2 single-screw extruder was utilized and the PLA–date pits pellets were introduced into it and the extruder facilitated the melting, extrusion, and formation of continuous composite filaments. The composite filaments were then fed into a Creality 3D Ender-3 V2 3D printer to fabricate the composite samples with the test samples’ standard dimensions. Figure 1 demonstrates the step-by-step production process involved in creating date pit powder, composite filaments, and 3D-printed samples. The figure showcases different stages of the production process with corresponding arrows or labels indicating the flow of production procedures. The test samples’ design was created in SolidWorks. The design file was then imported into Cura, a slicing software commonly used for 3D printing. After the modifications, the Cura software introduced the G-code used with the 3D printer. The printing speed was set at 50 mm/s, ensuring a consistent and efficient printing process. The printing density was maintained at 100% to achieve optimal structural integrity. The extruder temperature ranged from 200 to 210 °C, which was carefully controlled to ensure proper material flow and adhesion. Additionally, the bed temperature was kept constant at 70 °C to promote adhesion and minimize warping. All the settings of the 3D printer were held constant for all specimens to solely investigate the effect of adding DP powder into the PLA on the different characteristics of 3D-printed products besides the effect of the heat treatment process. The final produced composites were then inserted into a furnace for heat treatment process to be annealed at 100 °C. The heating rate was set at 5 degrees per minute to ensure a gradual and controlled temperature increase. Different holding times were implemented during the annealing process to investigate their effects on the material properties. The composite test specimens were prepared with weight fractions of DP and heat treated at different duration times, as shown in Table 1.

### 2.2. Characterization and Testing

The shore D technique was utilized to evaluate the PLA–DP composites’ hardness, based on ASTM D2240 [30]. The hardness were measured in six different regions along the surface of each composite specimen, and then the average was determined. Additionally, the synthesized samples were created following the guidelines of ISO 604 Plastics [31], L = 16 mm and Φ = 8 mm for the compression test, to gather information on the compressive mechanical properties. The test was carried out using an Instron 5582 micro tester with a 2 mm/min strain rate.

The Young’s modulus and ultimate compressive strength were obtained from the machine output diagram (stress vs. strain). To ensure the average value of each property, 5 specimens were fabricated and tested.

### 2.3. Finite Element Analysis (FEA)

**Figure 2:**
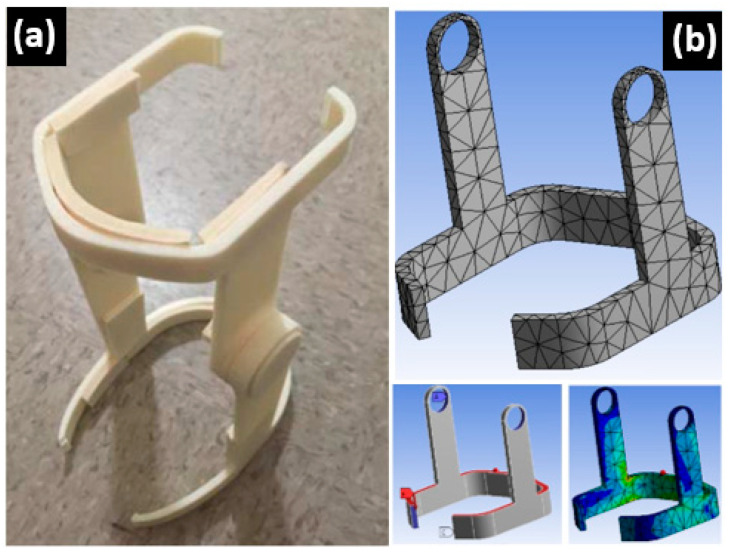
(**a**) Real part of knee orthotics, (**b**) Simulation model of lower brace of a knee orthotics.

The maximum ability for a material to withstand a load (load-carrying capacity) can be evaluated based on measuring the generated stresses on the surfaces during the loading process [32]. Consequently, it is necessary to measure the stress on contact during these processes to confirm the maximum capacity for the treated and untreated PLA–DP suggested composites in relation to human load. To accomplish this, an FEM model was constructed utilizing a static structure backage in ANSYS software for the lower brace of a knee orthotics from Hendricks et al. [33], as depicted in Figure 2. The model was created according to the mentioned study. The lower brace of a knee orthotic was made of a PLA–DP composite material based on the experimental measurements and inserted into the software. The lower brace model had meshed tetrahedron and hexahedron elements, and this resulted in creating 2164 nodes and the connection of nodes formed 863 elements. Figure 2 depicts the boundary conditions applied to lower brace model, based on the same study based on a human load. After the simulation, the highest level of equivalent and shear stresses were recorded.

### 2.4. Adaptive Neuro-Fuzzy Inference System (ANFIS)

**Figure 3:**
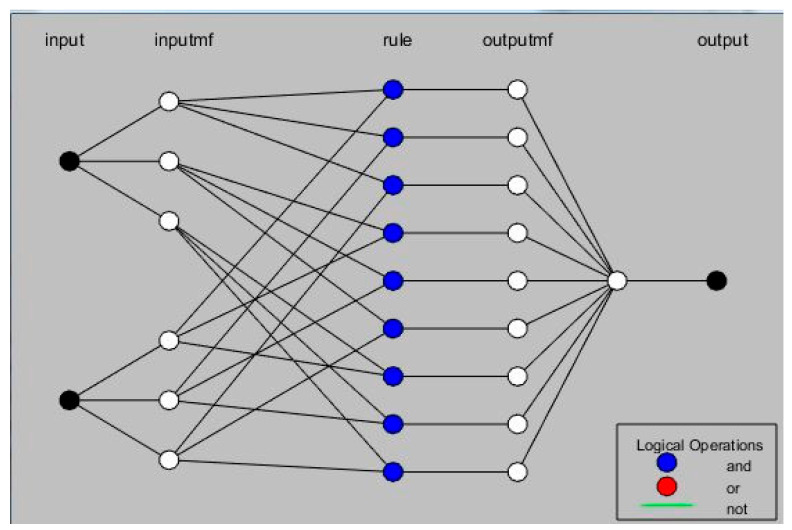
ANFIS architecture.

The ANFIS model combines an artificial intelligence technique called artificial neural networks (ANN) with fuzzy logic. Fuzzy logic helps convert qualitative inputs into clear outputs, but it has no specific approach for this conversion. In addition, it can take a long duration to adjust the membership functions. On the other hand, ANN has a strong ability to adapt to its environment. Therefore, by incorporating ANN, fuzzy logic systems can efficiently adapt fuzzy logic membership functions. The ANFIS model was created using a design tool in MATLAB called Adaptive Neuro-Fuzzy. This tool offers a user-friendly interface that allows for developing and assessing fuzzy systems utilizing a graphical user interface (GUI). To initiate the fuzzy interface system used for training, save the trained models, and illustrate them, a new Sugano system was loaded from the GUI menu bar. Figure 3 shows the ANFIS framework utilized in the current investigation. The model consists of two inputs (in the current study, DP weight fraction and heat treatment duration time) and three outputs (ultimate strength, Young’s modulus, and hardness), using 3 separated ANFIS models. The figure shows the use of three Gaussian membership functions for each input.

The ANFIS models typically consist of five layers which employ a hybrid learning approach to adapt to the input and output data. The first layer is called the fuzzification where each input node converts into a linguistic value using a membership function. Layer 2: The product layer contains non-adaptive nodes. Every node located in the second layer takes the input signals it receives and uses it to calculate the firing power of each rule utilizing the following equation. This layer produces the membership values product for the corresponding input. The third layer represents the normalization layer containing stationary nodes. Every individual node within this particular layer represents the normalized version of the intensity of firing from the preceding layer. Furthermore, every node obtains the intensity of firing from the product layer and proceeds to normalize it by dividing the value by the total sum of all firing strengths derived from the product layer. Implementing such a process guarantees that the collective sum of the normalized firing strengths amounts to 1. In the fourth layer, the de-fuzzification is conducted where it is designed to adapt and adjust to changing conditions. The final layer, known as the output layer, is responsible for producing the modeled result of the ANFIS network.

The ANFIS network was trained using a dataset that included input/output pairs necessary to create the model. This model uses a combination of different membership functions (MFs), rule bases, and parameters from the training dataset to map inputs to their corresponding outputs adaptively. In the current study, the experimental results were split into two parts: 90% of the data was allocated for training the ANFIS model. In contrast, the remaining 10% was used for testing the model’s performance. So, the model was initiated using the training experimental data, and the predicted mechanical characteristics were determined.

## 3. Results and Discussion

**Figure 4:**
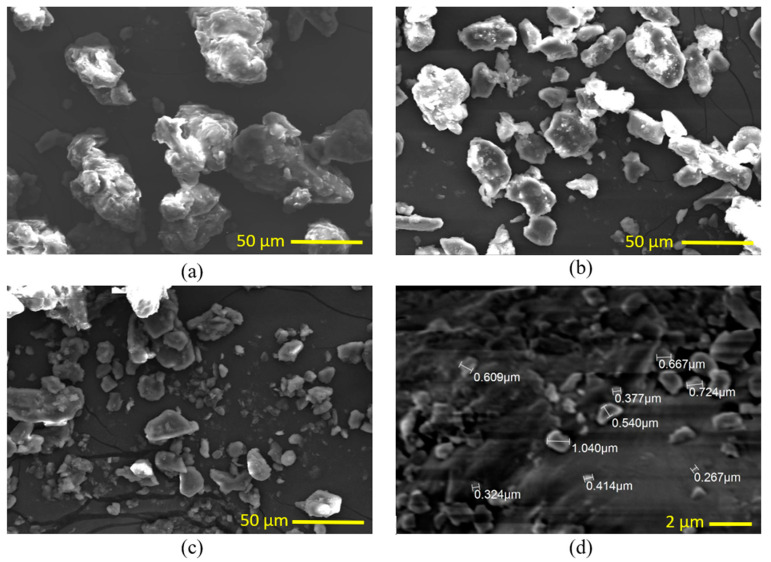
Date pit SEM images (**a**) after pulverization in a grain miller, and (**b–d**) after ball milling (**b**) 4 h, (**c**,**d**) 8 h.

In Section 2.1, the preparation of date pit powder, it is explained that the grinding process involves multiple stages. Figure 4 demonstrates the changes in the DP particles’ morphology throughout the production procedure. Figure 4a shows the particles after pulverization, although Figure 4b–d show the particles after the ball milling.

Figure 4a presents a scanning electron microscope image that provides the DP particles’ morphology subsequent to being processed inside the grain miller. The image reveals that the particles exhibit irregular equiaxed shapes and the roughness of the particles surface is high. Additionally, the particle size changes from 25 µm up to 50 µm. On the other hand, Figure 4b showcases the DP particles morphology after undergoing four hours of ball milling. As a result of the combined forces (shear and compressive) generated by the balls motion within the jar of the ball milling machine, the particles size reduces and becomes flat. Subsequently, Figure 4c,d present the DP particles morphology following eight hours of crushing inside the ball milling machine. The particle size is significantly reduced to approximately 0.6 µm, and the particles acquire a flaky shape.

**Figure 5:**
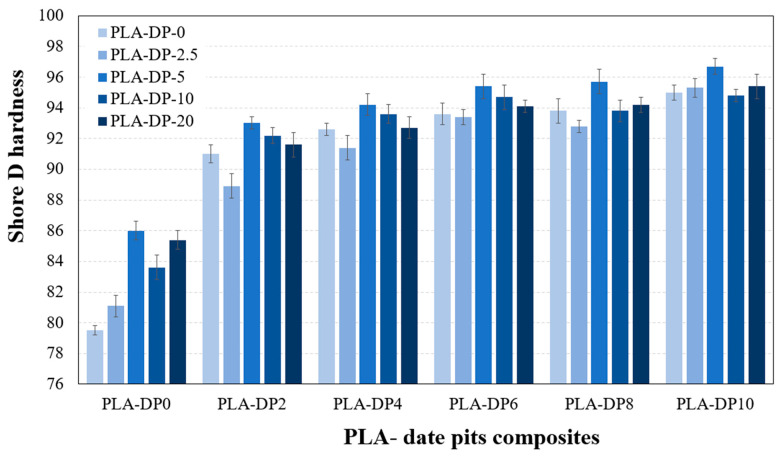
The hardness of 3D-printed PLA-DP composites at various annealing duration times.

Figure 5 shows how the hardness changes with variations in the DP concentration and the heat treatment duration time. The results indicate that increasing the concentration of the DP results in increased hardness, with PLA containing 10 wt.% of date pits showing the highest hardness with an increase of 20 % compared with pure PLA. The hardness of the produced composites is influenced by the intermolecular bonds established between the DP particles and the PLA matrix. A higher composite hardness suggests that the filler particles are homogeneously distributed throughout the polymeric matrix. This uniform distribution allows for an effective load transfer between the composite elements (PLA and DP particles) [34]. Therefore, based on the recorded results, it can be inferred that the DP particles are homogeneously distributed within the 3D-printed sample. In the hardness test, the heat treatment samples exhibited higher hardness than pure PLA for all heat treatment durations except for 2.5 h. To be more specific, the heat treatment at 110 °C for 5 h resulted in the highest hardness among all the samples. For example, the pure PLA had a hardness of 86 after 5 h of heat treatment, which was 8% higher than the untreated PLA. This enhancement was also observed in other PLA-date pit composites compared with specimens treated at different times. However, the hardness decreased with increasing duration time beyond 5 h. The samples treated for 2.5 h had hardness values close to those of the untreated samples. This could be attributed to the short duration of heat treatment, which might not have been sufficient to treat the samples’ upper layers. The hardness of the PLA composite samples decreased after heat treatment for 10 and 20 h. This decrease can be attributed to the degradation of the polymeric material that occurs under longer heat treatment durations [23]. In conclusion, annealing 3D-printed samples for 5 h under a temperature of 100 °C results in the most substantial enhancement in hardness.

**Figure 6:**
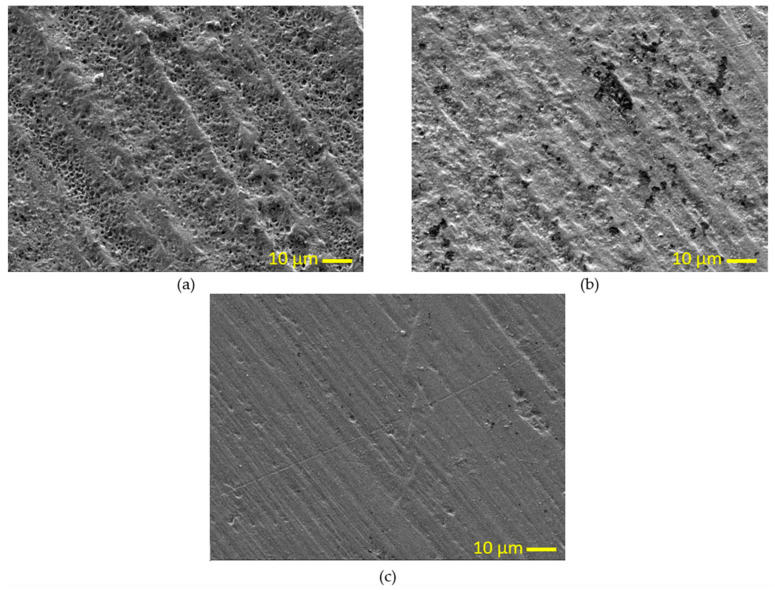
SEM images of PLA (**a**) without heat treatment, (**b**) after 2.5 h of treatment, and (**c**) after 5 h of treatment.

For a further demonstration of the annealing duration influence on PLA samples, scanning electron microscopy images for the samples’ surfaces are shown in Figure 6 where Figure 6a shows the surface before annealing, (b) after annealing for 2.5 h, and finally (c) after annealing for 5 h. The untreated samples exhibit numerous voids, and interference between the printed filaments is evident. With 2.5 h of heat treatment, some voids disappear while others coalesce, resulting in larger voids that weaken the surface of the samples. However, after 5 h, the voids disappear entirely, and the surface becomes smooth, which indicates a noticeable increase in hardness.

**Figure 7:**
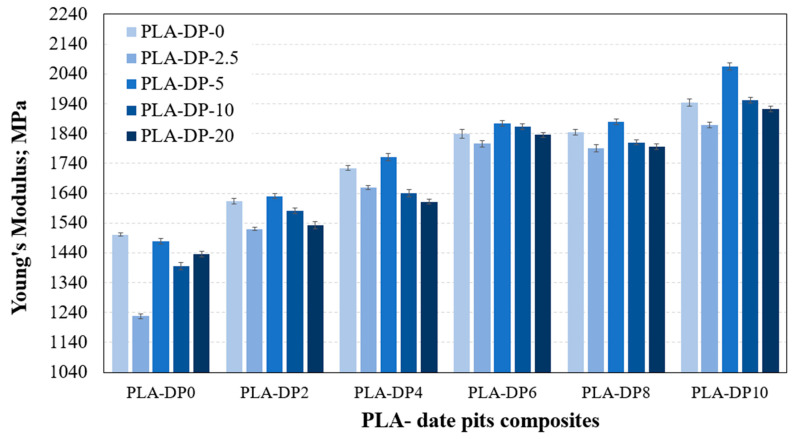
Young’s modulus of 3D-printed PLA–DP composites at various annealing duration times.

**Figure 8:**
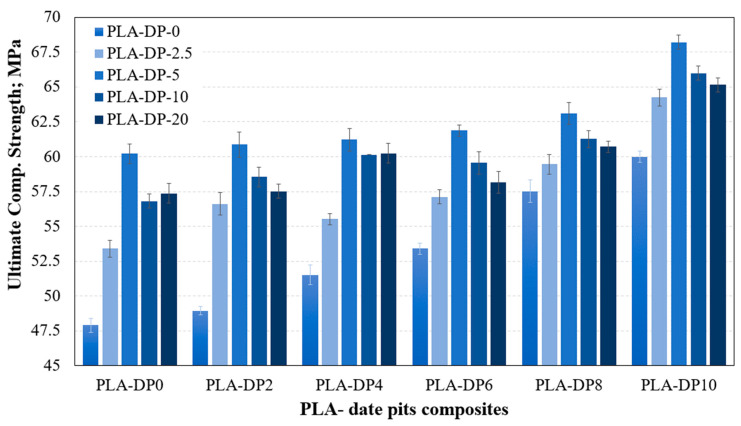
Ultimate compressive strength of 3D-printed PLA-DP composites at various annealing duration times.

To evaluate the effect of the incorporation of DP particles on the mechanical characteristics of PLA–DP composites, the composite samples were compressed and the results were recorded. The incorporation of DP into the PLA filament facilitated the dispersion of particles among the PLA chains. This resulted in a reduction in the flexibility of the PLA chains, leading to an improvement in the characteristics of the 3D-printed samples. Figure 7 and Figure 8 illustrate the change in the modulus of elasticity and the compressive strength for various PLA–DP compositions and annealing durations. As the concentration of DP increased, there was a corresponding increase in the modulus of elasticity. The pure PLA recorded 1501 MPa modulus of elasticity, which increased by 22% for 10 wt.% of date pits, reaching 1944 MPa. Moreover, increasing the DP concentration in the composite enhanced the composite strength, which was increased by 25% for 10 wt.% of date pits compared with pure PLA. The improvement in composite strength occurred due to the homogeneous dispersion of the DP particles within the PLA filament during the production process, which facilitated in absorbing the load dissipate. Additionally, the presence of filler particles prevented the crack propagation that occurred in the sample during the compression test [35]. This led to an increase in the overall compressive strength.

The untreated PLA specimens had an average modulus of elasticity and strength of 1501 MPa and 47.9 MPa, respectively. However, after 5 h of heat treatment, these values increased to 1541 MPa and 60.22 MPa, improving by 2.7% and 25.7%, respectively. Increasing the annealing duration beyond 5 h resulted in a decline in the characteristics of the PLA–DP samples. However, even after 20 h of heat treatment, the ultimate strength remained better than that of the untreated PLA samples, and the enhancement reached 19.77%. The main factor contributing to the improved characteristics of PLA–DP composites following the heat treatment process is the increase in crystallinity that occurs when the PLA is annealed at higher temperatures [36]. Nevertheless, it is noticeable that a decrease in mechanical characteristics occurred after heat treatment for more than 5 h, which can be attributed to the initiation of PLA degradation, as mentioned earlier.

**Figure 9:**
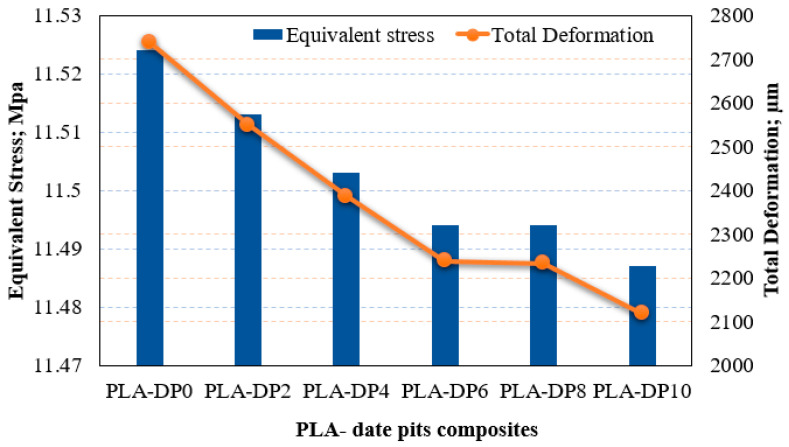
Equivalent von-Mises stress on the lower brace knee orthotics; different DP weight fraction.

**Figure 10:**
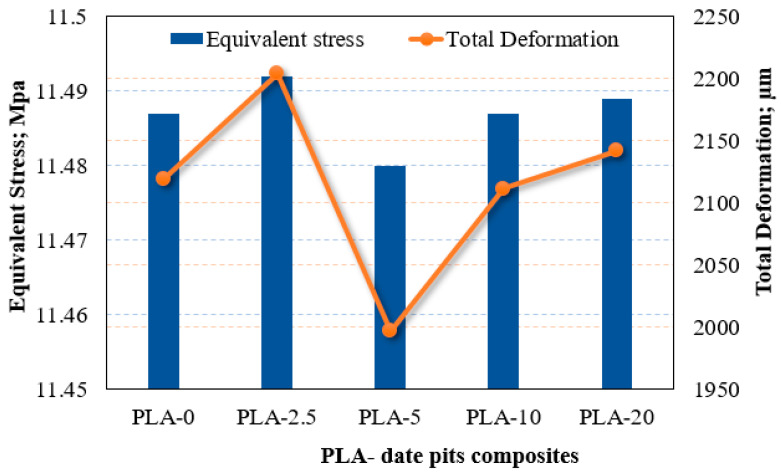
Equivalent von-Mises stress on the lower brace knee orthotics; different heat treatment duration times.

As previously discussed in Section 2.3, the load-carrying capacity of the material could be assessed by recognizing stress distribution across the prepared composite samples. The resulting stress generated on the lower brace of the knee orthotics for different DP concentrations and different annealing durations are shown in Figure 9 and Figure 10. It is obvious that raising the weight fraction of the DP resulted in a reduction in the generated stress on the lower brace knee orthotics. Additionally, composite samples that underwent a 5 h heat treatment exhibited the lowest stress values compared with the other composites. The finite element analysis findings support that the inclusion of date pit particles in the PLA, along with a 5 h heat treatment, enhances their load-carrying capacity. Furthermore, the finite element results illustrate that the total deformation decreased with the increase of date pit weight fraction and this decrease was more obvious after heat treatment of the samples for 5 h. In order to support this observation, we would like to refer you to the work of Mariam et al. [37] and Jayanth et al. [23] who conducted similar studies on the effect of heat treatment on PLA. Their findings align with our results, demonstrating that heat treatment can improve the mechanical properties of polymer composites, leading to reduced deformation and increased load-carrying capacity.

**Figure 11:**
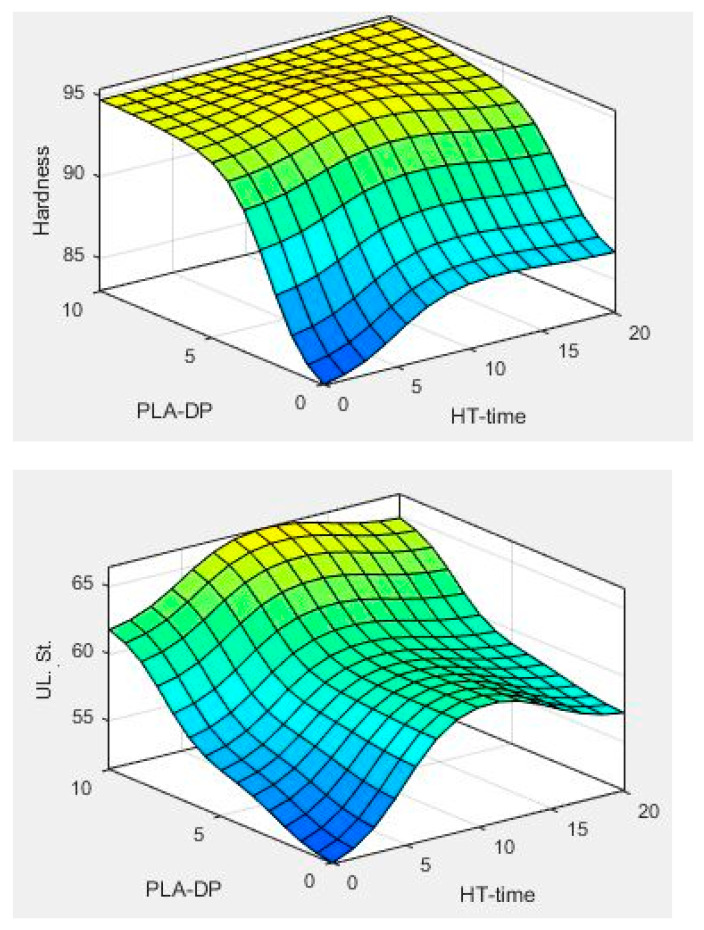

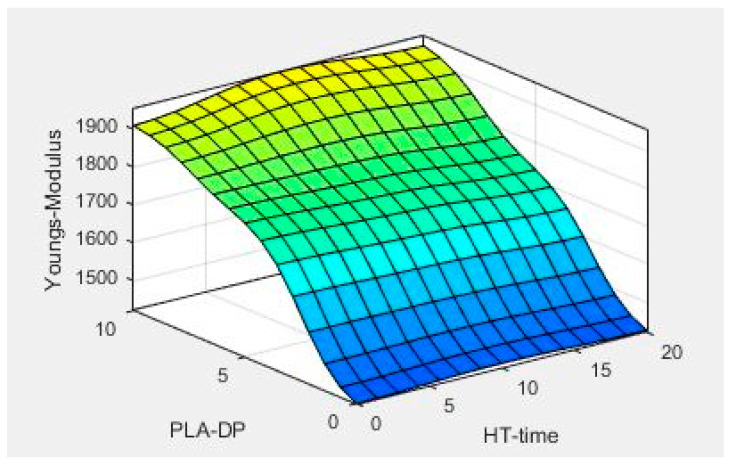
Hardness, modulus of elasticity, and strength in relation to change of DP weight fraction, heat treatment time.

The ANFIS model was employed to obtain the hardness, modulus of elasticity, and strength of the PLA–DP composites based on two input variables, date pit weight fraction (PLA/DP), and heat treatment duration time (HT). The impact of the two parameters on the output variation of hardness, the modulus of elasticity, and strength is presented in Figure 11.

Figure 11 indicates that the changing DP concentration and annealing duration time have a noticeable effect on the mechanical characteristics of the produced composite and are responsible for altering the surface contour of each property. Generally, increasing the DP concentration resulted in an enhancement in the mechanical properties of the PLA–DP composites, which is consistent with the experimental results. It is worth noting that the alteration of the DP concentration did not change the fact that annealing for 5 h corresponds to the optimal mechanical characteristics, which is also in line with the experimental outcomes.

**Figure 12:**
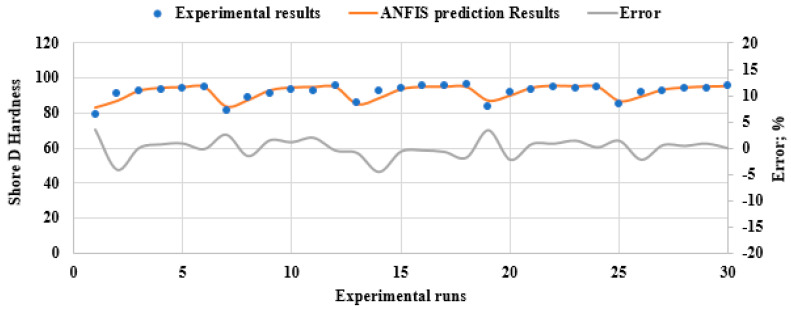

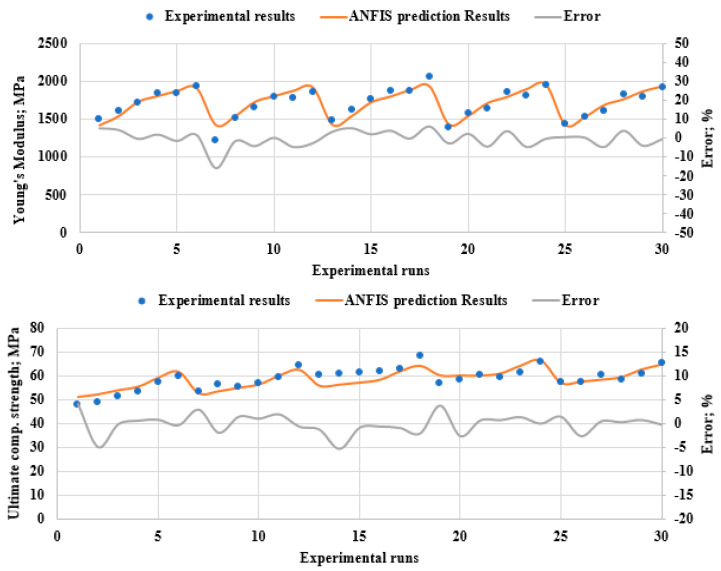
The comparison between experimental measured mechanical properties and ANFIS predicted ones for different DP wt.% and heat treatment time.

Figure 12 presents a comparison between the experimentally measured mechanical properties and the corresponding mechanical properties predicted by ANFIS. The X-axis represents the total number of experiments conducted on all samples under all conditions, including all variables. It provides an overview of the cumulative number of experiments performed throughout the study, while the Y-axis represents the properties that were measured experimentally or predicted using the ANFIS software. The points that exhibit a high degree of similarity with the experimental data are the points used for the training process. Other points represent the test data, which have a noticeable difference from the experimental data. However, the average percentage error between the predicted and experimental data for hardness, modulus of elasticity, and strength are less than 9.88e-3%, 0.18%, and 0.08%, indicating that the ANFIS model provides a reasonably accurate prediction of the PLA composite mechanical properties.

## 4. Conclusions

The estimation of mechanical properties for composite materials is a complex process that involves a non-linear procedure. To overcome such challenges, artificial intelligence approaches, such as ANFIS, have been developed specifically for modeling and predicting non-linear systems. This investigation proposes ANFIS-based estimation models to prognosticate the mechanical characteristics of 3D-printed PLA–DP composites that vary in DP concentrations, and under different annealing durations. The data used for model training was obtained through experimental testing. A comprehensive production process of the PLA green composite was conducted that involved a heat treatment to reduce residual stresses caused by the 3D-printing process. The PLA–DP composites were experimentally tested to determine their mechanical characteristics. An FEM was used to estimate load-carrying capacity based on surface stresses. The results showed that incorporating 10% DP particles increased the hardness, modulus of elasticity, and strength by 20%, 22%, and 25%, respectively. Furthermore, the heat treatment of the PLA–DP composites enhanced the mechanical properties by more than 25% for ultimate compressive strength. The FEA results demonstrated a reduction in generated stresses with the incorporation of DP particles, as well as after the heat treatment process, which reflects an improvement in the load-carrying capacity. Finally, the ANFIS model accurately predicted the mechanical characteristics of the PLA–DP composites under different conditions and a negligible average percentage error was recorded.

## Figures and Tables

**Table 1:** Detailed prepared samples.

Sample Denotation	Heat Treatment Duration Time (Hours)				
	PLA-DP-0	PLA-DP-2.5	PLA-DP-5	PLA-DP-10	PLA-DP-20
PLA-DP0	0 wt.%	0 wt.%	0 wt.%	0 wt.%	0 wt.%
	0 h	2.5 h	5 h	10 h	20 h
PLA-DP2	2 wt.%	2 wt.%	2 wt.%	2 wt.%	2 wt.%
	0 h	2.5 h	5 h	10 h	20 h
PLA-DP4	4 wt.%	4 wt.%	4 wt.%	4 wt.%	4 wt.%
	0 h	2.5 h	5 h	10 h	20 h
PLA-DP6	6 wt.%	6 wt.%	6 wt.%	6 wt.%	6 wt.%
	0 h	2.5 h	5 h	10 h	20 h
PLA-DP8	8 wt.%	8 wt.%	8 wt.%	8 wt.%	8 wt.%
	0 h	2.5 h	5 h	10 h	20 h
PLA-DP10	10 wt.%	10 wt.%	10 wt.%	10 wt.%	10 wt.%
	0 h	2.5 h	5 h	10 h	20 h

## Data Availability

Not applicable.
